# Discoid lupus erythematosus affecting the scalp

**DOI:** 10.1016/j.jdcr.2024.01.036

**Published:** 2024-03-16

**Authors:** Ilaria Scandagli, Elia Rosi, Antonella Di Cesare, Gianmarco Silvi, Giulia Nunziati, Prisca Guerra, Francesca Prignano

**Affiliations:** Department of Health Sciences, Section of Dermatology, University of Florence, Florence, Italy

**Keywords:** alopecia, discoid lupus erythematosus, follicular plugging

## Clinical presentation

**Fig 1:**
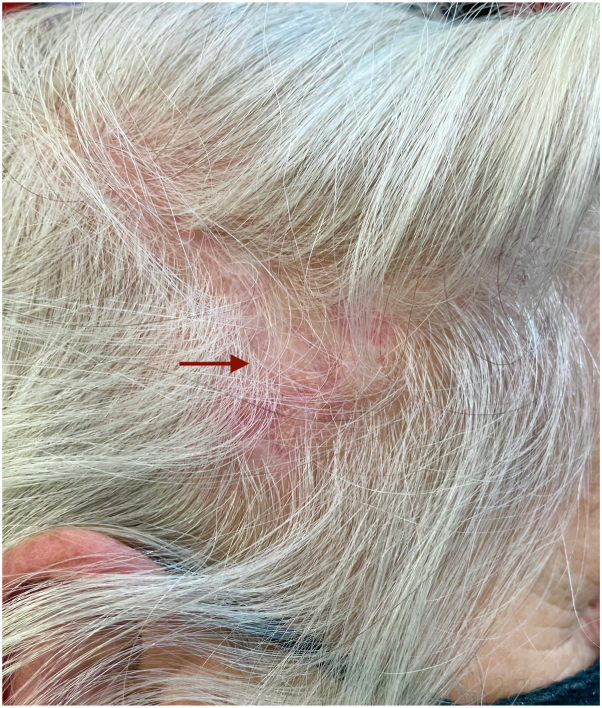
Red, scaly alopecic patch on her right parietal region.

A 57-year-old woman presented an erythematous-squamous alopecic patch on the right parietal region for 1 year (Fig 1). Her clinical history was notable for Hashimoto thyroiditis for 20 years.

## Dermatoscopic appearance

**Fig 2:**
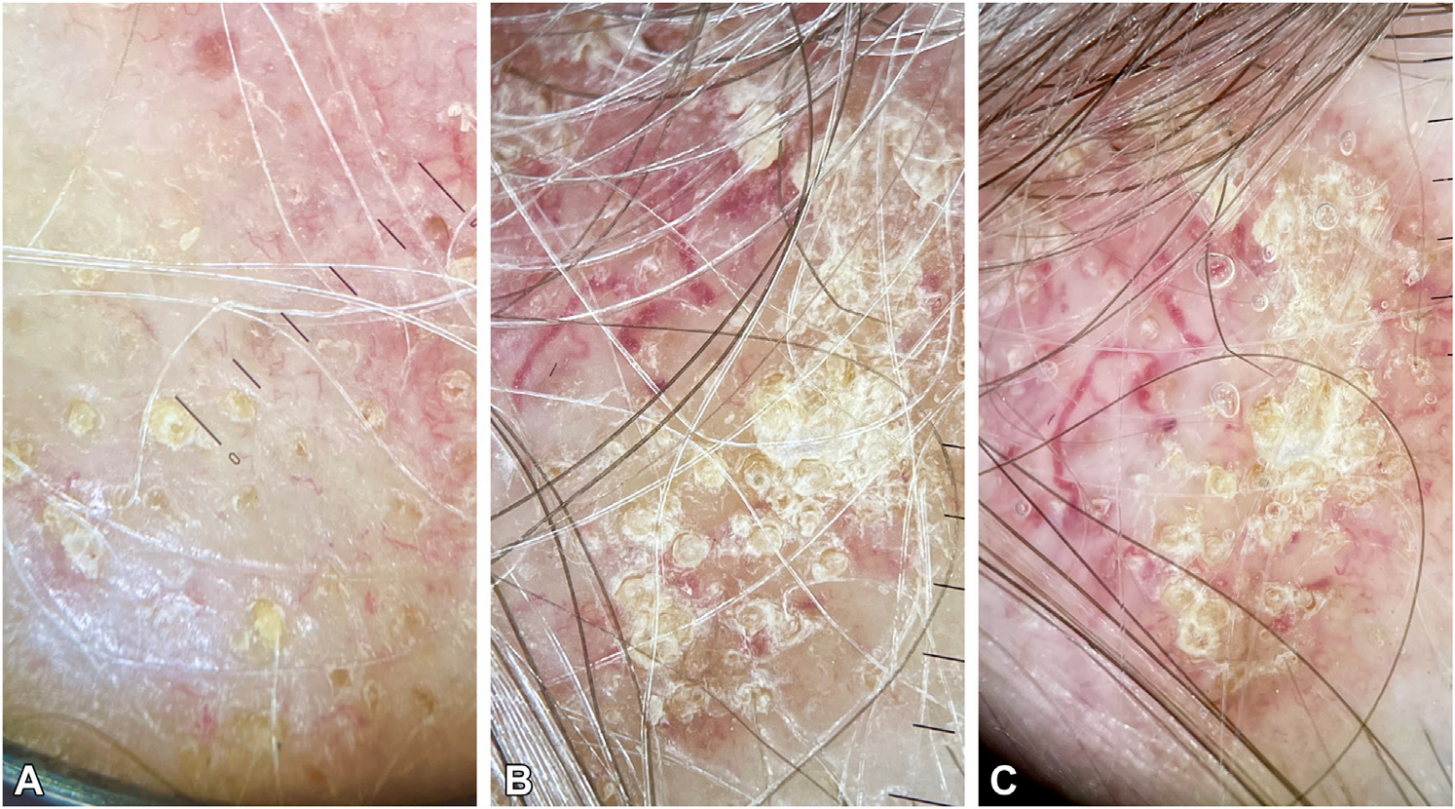
**A, B,** Trichoscopic features including a few yellow dots, keratotic follicular plugs, and white scaling between the follicles. **C,** Trichoscopy with immersion fluid showed thick branching vessels and white scarring patches.

Dry trichoscopy (Fig 2, *A* and *B*) revealed a few yellow dots, keratotic follicular plugs, and white interfollicular scaling. Trichoscopy with immersion fluid (Fig 2, *C*) highlighted thick arborizing vessels and white scarring areas.

## Histologic diagnosis

We performed a punch biopsy, and histopathologic examination showed a dense papillary and reticular lymphocytic infiltrate around the adnexa and vessels under an interface dermatitis; vacuolar degeneration and keratinocyte apoptosis were observed in the basal layer along with a thicker basement membrane and dilated blood vessels. Thus, a diagnosis of discoid lupus erythematosus (DLE) was made.

**Key message**

DLE is a primary lymphocytic cicatricial alopecia that primarily affects women in their 20s and 40s. It usually begins as round/oval purple patches that spread to erythematous alopecic areas with adherent scaling. As DLE progresses, these patches become fibrotic, atrophic, and pale, lacking follicular openings and often causing symptoms such as itching, distress, burning, and tenderness. In people with darker skin tones, DLE may lead to extensive depigmentation and the formation of completely depigmented alopecic areas. Dermatoscopy is an important tool for diagnosing DLE, particularly in lighter skin types from prevalent scarring alopecias such as lichen planopilaris. DLE typically presents larger yellow dots, keratotic follicular plugs, and thick arborizing vessels in active lesions.^1^

In contrast, lichen planopilaris shows smaller yellow dots, perifollicular scaling, and erythema. Dermatoscopy can provide important diagnostic clues that are not always visible clinically.^1^^,^^2^ However, skin biopsy remains the gold standard for a definitive diagnosis.

## Conflicts of interest

None disclosed.
